# Optimal contrast analysis with heterogeneous variances and budget concerns

**DOI:** 10.1371/journal.pone.0214391

**Published:** 2019-03-26

**Authors:** Show-Li Jan, Gwowen Shieh

**Affiliations:** 1 Department of Applied Mathematics, Chung Yuan Christian University, Taiwan, Republic of China; 2 Department of Management Science, National Chiao Tung University, Taiwan, Republic of China; Griffith University, AUSTRALIA

## Abstract

The omnibus test is commonly applied to evaluate the overall disparity between group means in ANOVA. Alternatively, linear contrasts are more informative in detecting specific pattern of mean differences that cannot be obtained via the omnibus test. This article concerns power and sample size calculations for contrast analysis with heterogeneous variances and budget concerns. Optimal allocation procedures for the Welch-Satterthwaite tests of standardized and unstandardized contrasts are presented to minimize the total sample size with the designated ratios, to meet a desirable power level for the least cost, and to attain the maximum power performance under a fixed cost. Currently available methods rely exclusively on simple allocation formula and direct rounding rule. The proposed allocation strategies combine the computing techniques of nonlinear optimization search and iterative screening process. Numerical assessments of a randomized control trial for the overcoming depression on the Internet are conducted to demonstrate and confirm that the approximate procedures do not guarantee optimal solution. The suggested approaches extend and outperform the existing findings in methodological soundness and overall performance. The corresponding computer algorithms are developed to implement the recommended power and sample size calculations for optimal contrast analysis.

## Introduction

Within the context of analysis of variance (ANOVA), the omnibus *F* test is widely used for detecting the overall mean differences. Alternatively, many important research questions may be formulated as a linear combination of the population group means. Hence, a *t* test of individual contrast provides much more information than an omnibus hypothesis in assessing particular relation between the mean effects. Comprehensive exposition and further information can be found in Kutner et al. [1] and Maxwell and Delaney [2]. However, it has been noted in many actual applications that the homogeneous variances assumption of ANOVA is frequently violated. For example, Grissom [3], Rosopa, Schaffer, and Schroeder [4], and Ruscio and Roche [5] stressed that variances can be extremely different across treatment groups in clinical and psychological study. To account for the impact of variance heterogeneity, the Welch–Satterthwaite procedure of Satterthwaite [6] and Welch [7] is commonly recommended as an alternative to the usual *t* test for detecting the substantive significance of a linear contrast. Accordingly, the contrast analysis under heterogeneity of variance is a generalization of the well-known Behrens–Fisher problem of testing the difference between two population means with unequal variances.

The general guidelines of experimental design and statistical analysis suggest that even the renowned test procedures do not warrant correct detection of treatment differences that are strongly expected or theoretically supported (Ioannidis [8], Moher, Dulberg, & Wells [9]). To prevent mistakenly dismissing an important contrast effect and to provide profound implications for ANOVA research, the underlying issues of power and sample size calculations must also be considered. It is prudent to emphasize that the traditional power and sample size procedures do not consider the cost implications. Notably, Allison et al. [10] advocated designing statistically powerful studies while minimizing costs. On the other hand, Marcoulides [11] emphasized the notion of maximizing power in designing studies under budget constraints. Although power and sample size procedures are available for Welch’s test [12] of the difference between two means, Luh and Guo [13] noted that cost issues have not been incorporated in the Welch–Satterthwaite test for contrast analysis. Accordingly, Luh and Guo [13] described a formula for efficient sample size allocation in two scenarios. The first scenario is attaining a designated power with the minimum total cost, and the second scenario is maximizing the statistical power for a designated total cost. The suggested optimal sample sizes have a ratio that is proportional to the product of the ratio of contrast coefficients and the ratio of standard deviations divided by the square root of the ratio of unit sampling costs. The particular method is a direct extension of the optimal sample size formula in Dette and Munk [14] and Pentico [15] for detecting the difference between two means under the normality assumption.

A standard normal distribution can be viewed as a *t* distribution with an infinite number of degrees of freedom. Despite this large-sample argument, the importance of a Student’s *t* distribution is well recognized in statistical applications, especially when the sample size is small. Note that the underlying notion of incorporating the cost concerns is because the time, money, and other resources are limited in all practical studies. The power and sample procedures [16–20] stress the theoretical principles of the approximate degree of freedom tests using estimated degrees of freedom. Specifically, power and sample size calculations for the Welch’s [21] omnibus test have been presented in Jan and Shieh [16] and Shieh and Jan [17, 18]. On the other hand, Shieh and Jan [19] considered the problem of power and sample size for the Welch–Satterthwaite test of linear contrasts, but they did not consider budget issues. The related results in Jan and Shieh [20] are restricted for designing 2 × 2 factorial studies while minimizing financial costs. Therefore, these optimal sample size methods did not cover all the cost schemes for contrast analysis. To our knowledge, there has been no other optimal cost and allocation investigations for the Welch–Satterthwaite test of contrast analysis except for Luh and Guo [13]. As a generalization of the results in [16–20], the present study focuses on optimal contrast analysis by implementing the distributional properties of the Welch–Satterthwaite *t* statistic in cost and allocation evaluations. Accordingly, in addition to being able to contribute to the methodological development and understanding of the approximate degrees of freedom test procedure, it also facilitates the pedagogical and numerical comparisons of the suggested approaches and the methods of Luh and Guo [13] for optimal sample size determinations.

In addition to the unstandardized contrasts, the effect size reporting and interpretation practices suggest that the standardized effect sizes are useful when comparing results from multiple studies using measurement instruments whose raw units are not directly comparable, such as Fritz, Morris, and Richler [22], Lakens [23], and Takeshima et al. [24]. Notably, standardized contrasts of treatment effects and corresponding effect sizes in ANOVA have been investigated by, among others, Olejnik and Algina [25], Rosenthal, Rosnow, and Rubin [26], and Steiger [27]. The prescribed sample size studies of linear contrasts did not include the more involved situations of standardized contrasts. Hence, it is of theoretical importance to extend the power and sample size calculations for conducting hypothesis testing of standardized contrasts.

In view of the importance of methodological justification and computational support, this article aims to present a systematic and thorough discussion for the Welch-Satterthwaite tests of standardized and unstandardized contrasts. Optimal allocation approaches are presented to minimize the total sample size with the designated ratios, to meet a desirable power level for the least cost, and to attain the maximum power performance under a fixed cost. An Internet depression intervention example is employed to demonstrate the features of the suggested approaches. To facilitate the recommended procedures in planning research designs, computer algorithms are offered for optimal power and sample size calculations with the designated allocation and cost schemes.

## Methods

### Linear contrasts

Consider the one-way ANOVA model in which *Y*_*ij*_ denotes the *j*th value of the response variable from the *i*th treatment group and the observations are assumed to be independent and normally distributed:

$$Y_{ij}\;\sim \;N(\mu_{i},\;\sigma_{i}^{2}),$$ (1)

where *μ*_*i*_ and $\sigma_{i}^{2}$ are unknown parameters, *i* = 1, …, *G* (≥ 2) and *j* = 1, …, *N*_*i*_. In addition to or instead of the omnibus test, questions regarding a particular pattern of group differences can be tested with a contrast of mean values.

A contrast is defined as a linear combination of mean parameters

$$\psi \;=\;\sum_{i=1}^{G}l_{i}\mu_{i},$$ (2)

where *l*_*i*_ are the linear coefficients with $\sum_{i=1}^{G}l_{i}\;=\;0$. With the model assumption defined in Eq 1, an unbiased estimator $\hat{\psi }$ for the contrast *ψ* is of the form

$$\hat{\psi }\;=\;\sum_{i=1}^{G}l_{i}{\overset{-}{Y}}_{i}$$ (3)

where ${\overset{-}{Y}}_{i}\;=\;\sum_{j=1}^{N_{i}}Y_{ij}/N_{i}$ is the *i*th group sample mean and is an unbiased estimator of *μ*_*i*_ for *i* = 1, …, *G*. Moreover, the contrast estimator $\hat{\psi }$ given in Eq 3 has the distribution

$$\hat{\psi }\;\sim \;N(\psi ,\;\omega^{2}),$$ (4)

where $\omega^{2}\;=\;Var(\hat{\psi })\;=\;\sum_{i=1}^{G}l_{i}^{2}{\sigma_{i}^{2}/N}_{i}$. An unbiased estimator ${\hat{\omega }}^{2}$ of *ω*^2^ can be readily obtained by replacing the variance $\sigma_{i}^{2}$ in *ω*^2^ with its unbiased estimator $S_{i}^{2}$:

$${\hat{\omega }}^{2}\;=\;\sum_{i=1}^{G}\frac{l_{i}^{2}S_{i}^{2}}{N_{i}},$$ (5)

where $S_{i}^{2}\;=\;\sum_{j=1}^{N_{i}}{\left(Y_{ij}-{\overset{-}{Y}}_{i}\right)}^{2}/(N_{i}-1)$ is the sample variance for *i* = 1,…, *G*.

#### Test for difference

To appraise a linear contrast of the mean effects in terms of the hypothesis

$$H_{0}:\;{\psi \;=\;\psi }_{0}\;versus\;H_{1}:\;{\psi \neq \psi }_{0},$$ (6)

the test statistic is of the form

$$T\;=\;\frac{\hat{\psi }-\psi_{0}}{\hat{\omega }},$$ (7)

where *ψ*_0_ is a constant. The Welch–Satterthwaite procedure suggests that under the null hypothesis *H*_0_: *ψ* = *ψ*_0_, the quantity *T* has a convenient approximate distribution

$$T\;\dot{\sim }\;t(\nu ),$$ (8)

where $\nu \;=\;{\left\{\sum_{i=1}^{G}l_{i}^{2}{\sigma_{i}^{2}/N}_{i}\right\}}^{2}/\{\sum_{i=1}^{G}l_{i}^{4}{\sigma_{i}^{4}/[N_{i}^{2}(N}_{i}-1)]\}$ and *t*(*v*) is a *t* distribution with degrees of freedom *v*. For inferential purposes, the degrees of freedom *v* is replaced by its counterpart $\hat{\nu }$ with direct substitution of $\left\{S_{1}^{2},\;\ldots ,S_{G}^{2}\right\}$ for $\left\{\sigma_{1}^{2},\;\ldots ,\sigma_{G}^{2}\right\}$ in *v*, where

$$\hat{\nu }\;=\;\frac{{\left\{\sum_{i=1}^{G}l_{i}^{2}{S_{i}^{2}/N}_{i}\right\}}^{2}}{\;\sum_{i=1}^{G}l_{i}^{4}{S_{i}^{4}/[N_{i}^{2}(N}_{i}-1)]\;}$$ (9)

The test rejects H_0_ at the significance level *α* if $\left|T|>t_{1-\alpha /2}(\hat{\nu }\right)$ where $t_{1-\alpha /2}(\hat{\nu })$ is the upper 100(*α*/2) percentile of the *t* distribution $t(\hat{\nu })$.

Moreover, with the same theoretical arguments and analytic derivations, it can be shown that the statistic *T* has the general approximate distribution

$$T\;\dot{\sim }\;t(\nu ,\Delta ),$$ (10)

where *t*(*ν*, Δ) is a noncentral *t* distribution with degrees of freedom *v* and noncentrality parameter

$$\Delta \;=\;\frac{\;\psi \;-\;\psi_{0\;}}{\omega }.$$ (11)

Also, the power function of the Welch–Satterthwaite test can be approximated by

$$\pi (\Delta )\;=\;P\{\left|t(\nu ,\Delta )|>t_{1-\alpha /2}(\nu \right)\}.$$ (12)

#### Test for noninferiority and superiority

In addition to the two-sided test of difference for a contrast, it is of clinical importance to test the hypotheses for noninferiority and superiority between mean effects (Laster & Johnson [28], Mulla et al. [29], Piaggio et al. [30], Scott [31]). The problem of testing noninferiority and superiority can be unified by the following hypotheses when larger values of *ψ* are better:

$$H_{0}:\;{\psi \leq \psi }_{0}\;versus\;H_{1}:\;{\psi >\psi }_{0}$$ (13)

where *ψ*_0_ is the non-inferiority or superiority threshold (Fleming et al. [32], Gayet-Ageron et al. [33], Gayet-Ageron et al. [34], Gladstone & Vach [35], Wien [36]). When *ψ*_0_ < 0, the rejection of the null hypothesis implies noninferiority against the reference margin. Whereas the rejection of the null hypothesis indicates superiority over the reference bound for *ψ*_0_ > 0. The upper one-sided test procedure rejects the null hypothesis at the significance level *α* if $T>t_{1-\alpha }(\hat{\nu })$ and the associated power function is expressed as

$$\pi (\Delta )\;=\;P\{\left|t(\nu ,\Delta )|>t_{1-\alpha }(\nu \right)\}.$$ (14)

Related points to consider on switching between superiority and non-inferiority can be found in the report of the Committee for Proprietary Medicinal Products [37], Ganju and Rom [38], Lewis [39], and Murray [40].

### Standardized contrasts

The usual linear contrast has advantages in understanding the meaning of effect size because the scale is the same as the original units of analysis. Alternatively, the standardized contrasts provide a natural interpretation of net effect that is critical to transform the magnitude of a treatment combination with respect to the metric of a response variable. A standardized contrast effect ψ* is defined as

$$\psi^{*}\;=\;\frac{\;\psi \;}{\omega^{*}},$$ (15)

where $\omega^{*2}\;=\;\sum_{i=1}^{G}l_{i}^{2}{\sigma_{i}^{2}/q}_{i}$ and *q*_*i*_ = *N*_*i*_/*N*_*T*_ for *i* = 1, …, *G*, and $N_{T}\;=\;\sum_{i=1}^{G}N_{i}$. To detect a standardized contrast effect, a slightly different statistic than *T* is considered:

$$T^{*}\;=\;\frac{\;\hat{\psi }\;}{\hat{\omega }}.$$ (16)

Also, T* has the general distribution

$$T^{*}\;\dot{\sim }\;t(\nu ,\Delta^{*}),$$ (17)

where $\Delta^{*}\;=\;N_{T}^{1/2}\psi^{*}$.

#### Test for difference

For assessing the standardized contrast effects in terms of the hypothesis

$$H_{0}:\;\psi^{*}\;=\;\psi_{0}^{*}\;versus\;H_{1}:\;\psi^{*}\neq \psi_{0,}^{*}$$ (18)

the test statistic T* has the distribution

$$T^{*}\dot{\sim }\;t(\nu ,\Delta_{0}^{*}),$$ (19)

where $\Delta_{0}^{*}\;=\;N_{T}^{1/2}\psi_{0}^{*}$ and $\psi_{0}^{*}$ is a constant. The null hypothesis is rejected at the significance level *α* if $T^{*}<t_{\alpha /2}(\hat{\nu },\Delta_{0}^{*})$ or $T^{*}>t_{1-\alpha /2}(\hat{\nu },\Delta_{0}^{*})$ where $t_{\alpha /2}(\hat{\nu },\Delta_{0}^{*})$ and $t_{1-\alpha /2}(\hat{\nu },\Delta_{0}^{*})$ are the lower and upper 100(*α*/2) percentiles of the noncentral *t* distribution $t(\hat{\nu },\Delta_{0}^{*})$, respectively. The corresponding power function is

$$\pi^{*}(\Delta^{*})\;=\;P\{t(\nu ,\Delta^{*})<t_{\alpha /2}(\nu ,\Delta_{0}^{*})\}+P\{t(\nu ,\Delta^{*})>t_{1-\alpha /2}(\nu ,\Delta_{0}^{*})\}.$$ (20)

#### Test for noninferiority and superiority

To perform the upper one-sided test for noninferiority and superiority in terms of

$$H_{0}:\;\psi^{*}\leq \psi_{0}^{*}\;versus\;H_{1}:\;\psi^{*}>\psi_{0}^{*},$$ (21)

the test procedure rejects H_0_ at the significance level *α* if $T^{*}>t_{1-\alpha }(\hat{\nu },\Delta_{0}^{*})$. Accordingly, the power function is defined as

$$\pi^{*}(\Delta^{*})=P\{t(\nu ,\Delta^{*})>t_{1-\alpha }(\nu ,\Delta_{0}^{*})\}.$$ (22)

The reference values $\psi_{0}^{*}$ need to be prudently selected to reflect the planned tests of noninferiority or superiority with appropriate magnitude and sign.

### Sample size calculations

During the planning stage of a research study, a question of essential interest is how many subjects are needed in order to have the desired power for conducting a scientifically meaningful analysis. To extend the applicability of contrast analysis, optimal sample size procedures are presented with respect to distinct allocation and cost concerns.

#### Sample size ratios are fixed

For advance planning of unstandardized and standardized contrast analysis, the prescribed power functions *π*(Δ) and π*(Δ*) can be employed to calculate the sample sizes {*N*_*i*_, *i* = 1, …, *G*} needed to attain the specified power 1 − *β* for the chosen significance level *α*, null values *ψ*_0_ and $\psi_{0}^{*}$, contrast coefficients {*l*_*i*_, *i* = 1, …, *G*}, and parameter values $\left\{\left(\mu_{i},\sigma_{i}^{2}\right),i=1,\ldots ,G\right\}$. However, it is prudent to consider the design structure with a priori chosen sample size ratios {*r*_1_, …, *r*_*G*_} where *r*_*j*_ = *N*_*j*_/*N*_*g*_ ≥ 1, *j* = 1, …, *G*, with the *g*th group has the smallest sample size *N*_*g*_. Note that the sample size calculations in Shieh and Jan [19] are only applicable to the tests of difference for conventional linear contrasts. Hence, they did not consider the tests for the standardized contrasts.

The cost and effort to treat a subject often vary with treatment groups and it is sensible for researchers to take into account budget and resource constraints in research design. The total cost of an ANOVA study can be represented by the overhead cost and sampling costs through the following simple cost function

$$C_{T}\;=\;c_{0}+\sum_{i=1}^{G}c_{i}N_{i},$$ (23)

where *c*_*O*_ is the fixed overhead cost associated with the study, and *c*_*i*_ reflects unit sampling cost of each subject in group *i* for *i* = 1, … *G*. Apparently, the cost assessment reduces to the evaluation of total number of subjects $C_{T}\;=\;N_{T}\;=\;\sum_{i=1}^{G}N_{i}$ when *c*_*O*_ = 0 and *c*_*i*_ = 1 for *i* = 1, …, *G*. Under cost and power considerations, the following two scenarios arise naturally in choosing the optimal sample sizes.

#### Target power is fixed and total cost needs to be minimized

Despite the simple linear form of the objective cost function, the optimization process involves the designated power function as a nonlinear constraint. Thus, a closed form solution rarely exists for most situations. With the specifications of the significance level *α*, the desired power level 1 − *β*, the null effect size, contrast coefficients, and the model parameters of group means and variance components, the suggested approach is composed of two key steps.

First, the preliminary set of sample sizes {*N*_*Pi*_, *i* = 1, …, *G*} for attaining the desired power performance while minimizing the total cost can be obtained with the NLPQN subroutine of the SAS/IML [41] package. However, the sample sizes are treated as continuous variables in the optimization process. The resulting sample sizes are most likely non-integer values. In view of the discrete nature of sample sizes, a systematic evaluation is conducted to find the proper result in the second step. The screening process of Shieh and Jan [18] is extended for a wider range of sample size combinations. Specifically, power calculations and cost assessments are performed for a total of 4^*G*^ sample size sets {*N*_*i*_, *i* = 1, …, *G*} with *N*_*i*_ = [*N*_*P*1_]– 1, [*N*_*P*1_], [*N*_*P*1_] + 1, or [*N*_*P*1_] + 2 for *i* = 1, … *G*, and [*M*] denotes the integer part of *M*. Then, the optimal allocation $\left\{N_{i}^{*},i=1,\ldots ,G\right\}$ is found through an inspection of the sample size combinations that attain the desired power while giving the least cost. If more than one set yields the same amount of least cost, the one giving the largest power is reported.

In contrast to the proposed thorough search, Luh and Guo [13] showed that the potential optimal sample size ratio for contrast analysis of unstandardized means is proportional to the product of the ratio of contrast coefficients and the ratio of standard deviations divided by the square root of the ratio of unit sampling costs:

$$\gamma_{i}\;=\;\frac{{\;N}_{i}\;}{N_{1}}\;=\;\frac{\;|l_{i}|\sigma_{i}c_{1}^{1/2}\;}{|l_{1}|\sigma_{1}c_{i}^{1/2}},\;i\;=\;1,\;\ldots ,\;G.$$ (24)

Note that the allocation ratios are derived with the standard normal *Z* statistic for known variances, rather than the *t* statistic under the assumption of unknown variances.

#### Total cost is fixed and actual power needs to be maximized

In addition to the prescribed design scheme, a problem of practical interest is to decide the best design in power performance when the total cost is fixed. Similar to the previous approach for optimal design, a two-step search procedure is performed. In this case, the specialized SAS/IML [41] NLPNRA subroutine is used to find the initial sample sizes {*N*_*P*1_, …, *N*_*PG*_} for the maximization of a nonlinear power function with the linear inequality cost constraint. The optimization algorithm assumes the sample sizes are continuous measurements and the computed outcomes {*N*_*P*1_, …, *N*_*PG*_} are extremely probable not integer values. To give the correct optimal solution, power and cost appraisals are performed in the second step for a total of 4^*G*^ sample size combinations {*N*_1_, …, *N*_*G*_} with *N*_*i*_ = [*N*_*P*1_]– 1, [*N*_*P*1_], [*N*_*P*1_] + 1, or [*N*_*P*1_] + 2 for *i* = 1, … *G*. Accordingly, the optimal allocation $\left\{N_{1}^{*},\ldots ,N_{G}^{*}\right\}$ is obtained through a detailed comparison of the sample size configurations that yields the greatest power while maintaining the restricted budget.

In this case, Luh and Guo [13] suggested the optimal sample size combination still has the allocation ratios given in Eq 24. The sample size of the first group is determined by $N_{1}\;=\;(C_{T}-c_{0})/(\sum_{i=1}^{G}c_{i}\gamma_{i})$ and the other sample sizes are then computed with *N*_*i*_ = *N*_1_*γ*_*i*_ for *i* = 2, …, *G*. It is unlikely that the sample sizes computed from the allocation ratios are whole numbers. The computed sample sizes need to be rounded up or down to the nearest integer and the outcomes are reported as the optimal sample sizes.

## Results

To explicate the usefulness of the recommended exact approaches and associated computer programs, the overcoming depression on the Internet (ODIN) study of Clarke et al. [42] is exemplified for power and sample size calculations. This research was a three-arm randomized control trial with a usual treatment control group and two ODIN intervention groups receiving reminders through postcards or brief telephone calls.

For demonstration, Luh and Guo [13] suggested a specific comparison of the two intervention programs to the usual treatment without access to ODIN with respect to the mental component summary scores at 16-week. The hypothesis testing is formulated as H_0_: *ψ* ≤ –4.2 versus H_1_: *ψ* > –4.2 with the linear coefficients {*l*_1_, *l*_2_, *l*_3_} = {0.5, 0.5, –1}. For the three study conditions of mail reminder, telephone reminder, and control group, {*N*_1_, *N*_2_, *N*_3_} = {75, 80, 100}, $\left\{{\bar{Y}}_{1},\;{\bar{Y}}_{2},\;{\bar{Y}}_{3}\right\}=\left\{34.7,32.3,35.5\right\}$, and $\left\{S_{1}^{2},\;S_{2}^{2},\;S_{3}^{2}\right\}=\left\{79.21,57.76,77.44\right\}$. It is shown that the contrast effect, estimated variance, and approximate degrees of freedom are $\hat{\psi }=–2$, ${\hat{\omega }}^{2}=1.2189$, and $\hat{\nu }=200.4582$, respectively. Moreover, the observed test statistic *T* = 1.9927 and the *p*-value = 0.0238. Hence, the test concludes that the contrast effect is significantly larger than –4.2 at α=0.05.

For the purposes of power analysis and sample size determination, the abovementioned findings are employed to provide planning values of the model parameters and design characteristics for upcoming Internet depression intervention study. With these parameter settings, contrast coefficients {0.5, 0.5, –1}, sample sizes {75, 80, 100}, and *ψ*_0_ = –4.2, the accompanying program shows that attained powers for the two-sided test and one-side test given in Eqs 6 and 13 are 0.5093 and 0.6335, respectively. The resulting powers are far less than the fairly common level of 0.80. Numerical computations reveal that the balanced group sample sizes of 183 and 144 are required to achieve the target power of 0.8 for the two-sided and one-side tests, respectively.

According to the cost-effectiveness study of Hollinghurst et al. [43], Luh and Guo [13] assumed that the fixed overhead cost *c*_*O*_ = 0 and the average operation costs {*c*_1_, *c*_2_, *c*_3_} = {20, 50, 100} as the unit sampling costs of the three treatment groups for future depression study. For the prescribed test for noninferiority, the approximate method of Luh and Guo [13] reported that the sample sizes {173, 93, 153} are required to attain the power performance of 0.80 with the least cost. Therefore, the total sample size and total cost are *N*_*T*_ = 419 and *C*_*T*_ = 23,410, respectively. It is also of fundamental interest to consider the optimal design problem in which the total number of subjects needs to be minimized. Luh and Guo [13] showed that the minimum sample sizes to assure the same power level of 0.80 are {98, 84, 193} with *N*_*T*_ = 375 and *C*_*T*_ = 25,460. Alternatively, the proposed approach suggests that the optimal sample sizes {173, 93, 152} and {97, 83, 193} are required to attain the designated power 0.80 with the least total cost and the smallest total sample size, respectively. The total sample size and total cost are *N*_*T*_ = 418 and *C*_*T*_ = 23,310 under the cost minimization consideration, whereas the corresponding results are *N*_*T*_ = 373 and *C*_*T*_ = 25,390 when minimum total sample size is desirable. The attained powers for the two sample size settings are 0.8000 and 0.8003, respectively, and they are nearly identical to the nominal level 0.80. For these two cases, Luh and Guo’s [13] method consistently gave greater total costs and larger total sample sizes than the suggested algorithm.

For the scenario of finding the optimal allocation to maximize power performance when the total cost is fixed as 22,000, the sample sizes computed by Luh and Guo [13] are {162.43, 87.73, 143.65}. They suggested finding the appropriate sample sizes by rounding up or down to the nearest integer. Accordingly, their chosen sample sizes are {162, 87, 144} with the total cost *C*_*T*_ = 21,990. When a computer is not available, the checking procedure entails laborious and tedious calculations especially for four or more groups. Instead, the optimal sample size allocation computed by the proposed approach is {160, 88, 144} which perfectly meets the planned budget. Moreover, exact computation shows that the resulting power 0.7795 of the optimal structure is larger than the power 0.7793 attained by the prescribed sample sizes {162, 87, 144}. Hence, the proposed algorithm is superior to the approximate procedure of Luh and Guo [13]. Generally, the computations of optimal solutions can be simplified by the approximate methods without much loss in accuracy, especially when the sample sizes are large. However, the proposed approaches will produce more accurate results across all sample sizes.

To demonstrate the hypothesis testing, power computation, and sample size determination for the standardized contrasts, the comparison of mental component summary scores between the depression interventions is analyzed next. It follows from the definitions of the unstandardized contrast *ψ* and the standardized contrast ψ* that a working value of $\psi_{0}^{*}$ is $\psi_{0}/{\left(N_{T}{\hat{\omega }}^{2}\right)}^{1/2}=-0.2382≐-0.25$. For simplicity’s sake, the null standardized effect is set as $\psi_{0}^{*}=-0.25$. Then, the hypothesis testing in terms of the standardized measure is formulated by H_0_: ψ* ≤ −0.25 versus H_1_: ψ* > –0.25. With the given data, the computations show that the standardized test statistic T* = –1.8115, the critical value *t*_0.95_(200.4582, –3.9922) = –2.3390, and the *p*-value = 0.0149. It is concluded that the standardized effect is significantly larger than –0.25 at *α* = 0.05.

With the parameter settings {*μ*_1_, *μ*_2_, *μ*_3_} = {34.7, 32.3, 35.5} and $\left\{\sigma_{1}^{2},\sigma_{2}^{2},\sigma_{3}^{2}\}\;=\;\{79.21,\;57.76,\;77.44\right\}$, the statistical power associated with the previous sample size combination {75, 80, 100} is 0.6988. For a balanced structure, it can be shown that the minimum sample size set {94, 94, 94} is necessary to attain the designated power of 0.8. In this case, the total sample size is *N*_*T*_ = 282 and the total cost is *C*_*T*_ = 15,980 for the fixed overhead cost *c*_*O*_ = 0 and the average operation costs {*c*_1_, *c*_2_, *c*_3_} = {20, 50, 100}. To attain the designated power 0.80 with the minimum total cost, the suggested procedure yields the optimal sample size scheme {305, 7, 15} with the total sample size *N*_*T*_ = 327 and total cost *C*_*T*_ = 7,950. On the other hand, the suggested allocation {73, 62, 143} incurs the least total sample size *N*_*T*_ = 278 with *C*_*T*_ = 18,860. In this case, the balanced design is not the optimal solution for either consideration of minimum total sample size or minimum total cost. When the maximum total cost is 22,000, the proposed optimal sample size structure is {815, 22, 46} with *N*_*T*_ = 883 and *C*_*T*_ = 22,000.

Note that all the numerical results of the optimal power and sample size procedures were computed with the supplemental SAS/IML algorithms. For ease of application, two different sets of computer programs are presented for the standardized and unstandardized contrast analysis.

## Conclusions and discussion

The Welch–Satterthwaite statistic and the associated approximate *t* distribution have an important utility in accommodating the impact of heterogeneity of variance in statistical inference. The technical account of diverse hypothesis-testing frameworks enhances the theoretical implication and practical usefulness of the Welch–Satterthwaite test for contrast analysis in the detection of difference or inferiority/superiority. Moreover, the integrated document of different contrast effect sizes facilitates the reporting and interpretation of important finding in standardized measure scaled by the associated variabilities and design characteristics or in simple magnitude expressed in the same metric as the original units of analysis. One important implication of this research is that the essence of the Welch–Satterthwaite procedure is properly recognized in related power and sample size calculations without a normal simplification. Nonlinear optimization routines and systematic numerical evaluations are synthesized to give optimal sample size allocations for contrast analysis. According to the analytic examination and numerical assessment, the suggested procedures ultimately outperform the existing sample size methods based on the normal approximation and integer rounding. Essentially, the collection of computer programs covers both the two-sided and one-sided hypothesis testing for the two distinct formulations of standardized and unstandardized contrasts. The presented appraisals of statistical power, sample size, and financial budget should be useful for researchers to justify their allocation strategy and project support in planning research design.

The general formulation of a linear contrast of group means permits a wide range of research hypotheses to be tested in ANOVA. To enhance the usefulness of contrast analysis under heterogeneity of variance, this article addresses the problem of optimal sample size calculations for the Welch–Satterthwaite test with cost constraints. The present study has three essential features. First, the two-sided and one-sided test procedures are presented for both the standardized and unstandardized contrasts in ANOVA under the heterogeneous variances assumption. Second, optimal sample size approaches are proposed for the two essential problems that when the target power is fixed and total cost needs to be minimized and when the total cost is fixed and actual power needs to be maximized. Third, computer codes are presented to implement the power and sample size computations of the Welch–Satterthwaite procedures. In sum, this study contributes to the current literature for optimal research designs by alleviating the limitations of existing investigations and extending the usefulness of contrast analysis in ANOVA under variance heterogeneity.

## Supporting information

S1 File SAS/IML programs for performing the tests of linear contrast. (PDF)

S2 File SAS/IML programs for performing the tests of standardized contrast. (PDF)
