# Supplementary material for: Optimal contrast analysis with heterogeneous variances and budget concerns
Source: PLoS One. 2019 Mar 26;14(3):e0214391. doi: 10.1371/journal.pone.0214391 (PMC6435144; doi:10.1371/journal.pone.0214391)
Supplement: S1 File — (PDF) [file pone.0214391.s001.pdf]

Supplement A1

SAS/IML program for performing the test of linear contrast

```
PROC IML;
*USER SPECIFICATIONS;
*TYPE I ERROR;ALPHA=0.05;
*GROUP MEANS;MEVEC={34.7 32.3 35.5};
*GROUP VARAINCES;S2VEC={79.21 57.76 77.44};
*CONTRAST COEFFICIENTS;LVEC={0.5 0.5 -1};
*NULL VALUE;PSI0=-4.2;
*SAMPLE SIZES;NVEC={75 80 100};
*END OF SPECIFICATIONS;

PSIH=LVEC*MEVEC`;
VARPSIH=SUM((LVEC##2)#S2VEC/NVEC);
STDPSIH=SQRT(VARPSIH);
TH=(PSIH-PSI0)/STDPSIH;
KH=(LVEC##2)#S2VEC/NVEC;
DFAH=SUM(KH)##2;
DFBH=SUM((KH##2)/(NVEC-1));
DFVTH=DFAH/DFBH;
CRITT=TINV(1-ALPHA/2,DFVTH);
CRITL=TINV(ALPHA,DFVTH);
CRITU=TINV(1-ALPHA,DFVTH);
PVALUET=2#SDF('T',TH,DFVTH);
PVALUEO=SDF('T',ABS(TH),DFVTH);

PRINT PSIH[FORMAT=8.4] VARPSIH[FORMAT=8.4] STDPSIH[FORMAT=8.4]
TH[FORMAT=8.4] DFVTH[FORMAT=8.4];
PRINT PSI0[FORMAT=8.4];
PRINT 'TWO-SIDED TEST' TH[FORMAT=8.4] CRITT[FORMAT=8.4] ALPHA
PVALUET[FORMAT=8.4] ;
IF TH<0 THEN PRINT ' LEFT-SIDED TEST' TH[FORMAT=8.4] CRITL ALPHA
PVALUEO[FORMAT=8.4];
ELSE PRINT 'RIGHT-SIDED TEST' TH[FORMAT=8.4] CRITU[FORMAT=8.4]
ALPHA PVALUEO[FORMAT=8.4];
QUIT;
```

Supplement A2

SAS/IML program for calculating the achieved power of the test of linear contrast

```
PROC IML;
*USER SPECIFICATIONS;
*TYPE I ERROR;ALPHA=0.05;
*GROUP MEANS;MUVEC={34.7 32.3 35.5};
*GROUP VARAINCES;VARVEC={79.21 57.76 77.44};
*CONTRAST COEFFICIENTS;LVEC={0.5 0.5 -1};
*NULL VALUE;PSI0=-4.2;
*SAMPLE SIZES;NVEC={144 144 144};NVEC={183 183 183};NVEC={173 93
153};
*NVEC={75 80 100};NVEC={162 87 144};
*END OF SPECIFICATIONS;

G=NCOL(VARVEC);
PSI1=LVEC*MUVEC`;PSID=PSI1-PSI0;
PRINT G ALPHA PSI1 PSI0 PSID;
PRINT MUVEC[FORMAT=8.4];PRINT VARVEC[FORMAT=8.4];
PRINT LVEC[FORMAT=8.4];PRINT NVEC;
VARPSI=(LVEC##2)*(VARVEC/NVEC)`;
DELTA=PSID/SQRT(VARPSI);
KV=(LVEC##2)#VARVEC/NVEC;V1=SUM(KV)##2;
V2=SUM((KV##2)/(NVEC-1));
DFVAP=V1/V2;
CRITT=TINV(1-ALPHA/2,DFVAP);
POWERT=CDF('T',-CRITT,DFVAP,DELTA)+SDF('T',CRITT,DFVAP,DELTA);
CRITL=TINV(ALPHA,DFVAP);CRITU=TINV(1-ALPHA,DFVAP);
IF DELTA<0 THEN POWERO=CDF('T',CRITL,DFVAP,DELTA);
ELSE POWERO=SDF('T',CRITU,DFVAP,DELTA);
NT=SUM(NVEC);
PRINT NT POWERT[FORMAT=10.6] POWERO[FORMAT=10.6];
QUIT;
```

Supplement A3

SAS/IML program for calculating the optimal sample sizes of the test of linear contrast when the sample size ratios are fixed

```
PROC IML;
*USER SPECIFICATIONS;
*TYPE I ERROR;ALPHA=0.05;
*DESIGNATED POWER;POWER=0.80;
*GROUP MEANS;MUVEC={34.7 32.3 35.5};
*GROUP VARAINCES;VARVEC={79.21 57.76 77.44};
*CONTRAST COEFFICIENTS;LVEC={0.5 0.5 -1};
*NULL VALUE;PSI0=-4.2;
*SAMPLE SIZE RATIOS;RVEC={1 1 1};
*END OF SPECIFICATIONS;

G=NCOL(VARVEC);
PSI1=LVEC*MUVEC`;PSID=PSI1-PSI0;
PRINT G ALPHA POWER PSI1 PSI0 PSID;
PRINT MUVEC[FORMAT=8.4];PRINT VARVEC[FORMAT=8.4];
PRINT LVEC[FORMAT=8.4];PRINT RVEC;
*TWO-SIDED TEST;N=2;
DO UNTIL (POWERT>POWER);
N=N+1;
NVEC=N#RVEC;NT=SUM(NVEC);
VARPSI=(LVEC##2)*(VARVEC/NVEC)`;
DELTA=PSID/SQRT(VARPSI);
KV=(LVEC##2)#VARVEC/NVEC;V1=SUM(KV)##2;
V2=SUM((KV##2)/(NVEC-1));
DFVAP=V1/V2;
CRITT=TINV(1-ALPHA/2,DFVAP);
POWERT=CDF('T',-CRITT,DFVAP,DELTA)+SDF('T',CRITT,DFVAP,DELTA);
END;
PRINT 'TWO-SIDED TEST' POWERT[FORMAT=8.4] NT NVEC;
*ONE-SIDED TEST;N=2;
DO UNTIL (POWERO>POWER);
N=N+1;
NVEC=N#RVEC;NT=SUM(NVEC);
VARPSI=(LVEC##2)*(VARVEC/NVEC)`;
DELTA=PSID/SQRT(VARPSI);
```

```
KV=(LVEC##2)#VARVEC/NVEC;V1=SUM(KV)##2;
V2=SUM((KV##2)/(NVEC-1));
DFVAP=V1/V2;
CRITL=TINV(ALPHA,DFVAP);
CRITU=TINV(1-ALPHA,DFVAP);
IF DELTA<0 THEN POWERO=CDF('T',CRITL,DFVAP,DELTA);
ELSE POWERO=SDF('T',CRITU,DFVAP,DELTA);
END;
PRINT 'ONE-SIDED TEST' POWERO[FORMAT=8.4] NT NVEC;
QUIT;
```

Supplement A4

SAS/IML program for calculating the optimal sample sizes of the test of linear contrast to meet a designated power level for the least cost

```
PROC IML;
*USER SPECIFICATIONS;
*TYPE I ERROR;ALPHA=0.05;
*DESIGNATED POWER;POWER=0.80;
*GROUP MEANS;MUVEC={34.7 32.3 35.5};
*GROUP VARAINCES;VARVEC={79.21 57.76 77.44};
*CONTRAST COEFFICIENTS;LVEC={0.5 0.5 -1};
*NULL VALUE;PSI0=-4.2;
*UNIT COSTS;CVEC={20 50 100};CVEC={1 1 1};
*END OF SPECIFICATIONS;

PSI1=LVEC*MUVEC`;PSID=PSI1-PSI0;
PRINT ALPHA POWER MUVEC;PRINT PSI1 PSI0 PSID;
PRINT VARVEC;PRINT LVEC;PRINT CVEC;

START C(NVEC) GLOBAL(CVEC);
TC=CVEC*NVEC`;
RETURN(TC);FINISH;

START PT(NVEC) GLOBAL(LVEC, VARVEC, PSID, ALPHA, POWER);
NT=SUM(NVEC);
VARPSI=(LVEC##2)*(VARVEC/NVEC)`;
DELTA=PSID/SQRT(VARPSI);
KV=(LVEC##2)#VARVEC/NVEC;V1=SUM(KV)##2;
V2=SUM((KV##2)/(NVEC-1));DFVAP=V1/V2;
CRITT=TINV(1-ALPHA/2,DFVAP);
POWERT=CDF('T',-CRITT,DFVAP,DELTA)+SDF('T',CRITT,DFVAP,DELTA);
P=POWER-POWERT;
RETURN(P);FINISH;

START PO(NVEC) GLOBAL(LVEC, VARVEC, PSID, ALPHA, POWER);
NT=SUM(NVEC);
VARPSI=(LVEC##2)*(VARVEC/NVEC)`;
DELTA=PSID/SQRT(VARPSI);
KV=(LVEC##2)#VARVEC/NVEC;V1=SUM(KV)##2;
```

```
V2=SUM((KV##2)/(NVEC-1));DFVAP=V1/V2;
CRITL=TINV(ALPHA,DFVAP);CRITU=TINV(1-ALPHA,DFVAP);
IF DELTA<0 THEN POWERO=CDF('T',CRITL,DFVAP,DELTA);
ELSE POWERO=SDF('T',CRITU,DFVAP,DELTA);
P=POWER-POWERO;
RETURN(P);FINISH;

G=NCOL(MUVEC);
OPTN=J(1,11,.);OPTN[1]=0;OPTN[2]=0;OPTN[10]=1;OPTN[11]=1;
MINNVEC=J(1,G,3.1);
BLC=MINNVEC//J(1,G,.);

CALL NLPQN(RC,XRT,"C",MINNVEC,OPTN,BLC) NLC="PT";
A=T(-1:2);MAT=J(4##G,G,0);
DO I=1 TO G;Z=1;M=J(4,G,1);M[,I]=A;DO J=1 TO
G;Z=Z@M[,J];END;MAT[,I]=Z;END;
MINVEC=FLOOR(XRT);
PVEC=J(NROW(MAT),1,0);NMAT=J(NROW(MAT),G,0);
DO I=1 TO NROW(MAT);
NVEC=MINVEC+MAT[I,];NMAT[I,]=NVEC;
NT=SUM(NVEC);
VARPSI=(LVEC##2)*(VARVEC/NVEC)`;
DELTA=PSID/SQRT(VARPSI);DF=NT-G;DFVEC=NVEC-1;
KV=(LVEC##2)#VARVEC/NVEC;V1=SUM(KV)##2;
V2=SUM((KV##2)/(NVEC-1));DFVAP=V1/V2;
CRITT=TINV(1-ALPHA/2,DFVAP);
POWERT=CDF('T',-CRITT,DFVAP,DELTA)+SDF('T',CRITT,DFVAP,DELTA);
PVEC[I,1]=POWERT;
END;
TVEC=NMAT*CVEC`;
LOC=LOC(PVEC>=POWER);
N2MAT=NMAT[LOC,];P2VEC=PVEC[LOC,];T2VEC=TVEC[LOC,];
T2MIN=T2VEC[><,];MINIVEC=LOC(T2VEC=T2MIN);
N2MINMAT=N2MAT[MINIVEC,];
P2MINVEC=P2VEC[MINIVEC,1];
T2MINVEC=T2VEC[MINIVEC,1];
POMAXMIN=P2MINVEC[<>,1];MAXMINI=P2MINVEC[<:>,1];
NMAXMIN=N2MINMAT[MAXMINI,];TOTALN=SUM(NMAXMIN);
OPTIMALN=NMAXMIN;POWERT=POMAXMIN;TOTALCOST=T2MIN;
```

```
PRINT 'TWO-SIDED TEST' OPTIMALN POWERT[FORMAT=7.4] TOTALCOST
TOTALN;

CALL NLPQN(RC,XRO,"C",MINNVEC,OPTN,BLC) NLC="PO";
A=T(-1:2);MAT=J(4##G,G,0);
DO I=1 TO G;Z=1;M=J(4,G,1);M[,I]=A;DO J=1 TO
G;Z=Z@M[,J];END;MAT[,I]=Z;END;
MINVEC=FLOOR(XRO);
PVEC=J(NROW(MAT),1,0);NMAT=J(NROW(MAT),G,0);
DO I=1 TO NROW(MAT);
NVEC=MINVEC+MAT[I,];NMAT[I,]=NVEC;
NT=SUM(NVEC);
VARPSI=(LVEC##2)*(VARVEC/NVEC)`;
DELTA=PSID/SQRT(VARPSI);DF=NT-G;DFVEC=NVEC-1;
KV=(LVEC##2)#VARVEC/NVEC;V1=SUM(KV)##2;
V2=SUM((KV##2)/(NVEC-1));DFVAP=V1/V2;
CRITL=TINV(ALPHA,DFVAP);CRITU=TINV(1-ALPHA,DFVAP);
IF DELTA<0 THEN POWERO=CDF('T',CRITL,DFVAP,DELTA);
ELSE POWERO=SDF('T',CRITU,DFVAP,DELTA);
PVEC[I,1]=POWERO;
END;
TVEC=NMAT*CVEC`;
LOC=LOC(PVEC>=POWER);
N2MAT=NMAT[LOC,];P2VEC=PVEC[LOC,];T2VEC=TVEC[LOC,];
T2MIN=T2VEC[><,];MINIVEC=LOC(T2VEC=T2MIN);
N2MINMAT=N2MAT[MINIVEC,];
P2MINVEC=P2VEC[MINIVEC,1];
T2MINVEC=T2VEC[MINIVEC,1];
POMAXMIN=P2MINVEC[<>,1];MAXMINI=P2MINVEC[<:>,1];
NMAXMIN=N2MINMAT[MAXMINI,];TOTALN=SUM(NMAXMIN);
OPTIMALN=NMAXMIN;POWERO=POMAXMIN;TOTALCOST=T2MIN;
PRINT 'ONE-SIDED TEST' OPTIMALN POWERO[FORMAT=7.4] TOTALCOST
TOTALN;
***********************************************;
QUIT;
```

Supplement A5

SAS IML program for calculating the optimal sample sizes of the test of linear contrast to attain maximum power performance for a fixed cost

```
PROC IML;
*USER SPECIFICATIONS;
*TYPE I ERROR;ALPHA=0.05;
*DESIGNATED POWER;POWER=0.80;
*GROUP MEANS;MUVEC={34.7 32.3 35.5};
*GROUP VARAINCES;VARVEC={79.21 57.76 77.44};
*CONTRAST COEFFICIENTS;LVEC={0.5 0.5 -1};
*NULL VALUE;PSI0=-4.2;
*UNIT COSTS;CVEC={20 50 100};*CVEC={1 1 1};
*TOTAL COST;COST=22000;
*END OF SPECIFICATIONS;

PSI1=LVEC*MUVEC`;PSID=PSI1-PSI0;G=NCOL(MUVEC);
PRINT ALPHA COST MUVEC;PRINT PSI1 PSI0 PSID;PRINT VARVEC;
PRINT LVEC;PRINT CVEC;

START PT(NVEC) GLOBAL(LVEC, VARVEC, PSID, ALPHA, POWER);
NT=SUM(NVEC);
VARPSI=(LVEC##2)*(VARVEC/NVEC)`;
DELTA=PSID/SQRT(VARPSI);
KV=(LVEC##2)#VARVEC/NVEC;V1=SUM(KV)##2;
V2=SUM((KV##2)/(NVEC-1));DFVAP=V1/V2;
CRITT=TINV(1-ALPHA/2,DFVAP);
POWERT=CDF('T',-CRITT,DFVAP,DELTA)+SDF('T',CRITT,DFVAP,DELTA);
RETURN(POWERT);FINISH;

START PO(NVEC) GLOBAL(LVEC, VARVEC, PSID, ALPHA, POWER);
NT=SUM(NVEC);
VARPSI=(LVEC##2)*(VARVEC/NVEC)`;
DELTA=PSID/SQRT(VARPSI);
KV=(LVEC##2)#VARVEC/NVEC;V1=SUM(KV)##2;
V2=SUM((KV##2)/(NVEC-1));DFVAP=V1/V2;
CRITL=TINV(ALPHA,DFVAP);CRITU=TINV(1-ALPHA,DFVAP);
IF DELTA<0 THEN POWERO=CDF('T',CRITL,DFVAP,DELTA);
ELSE POWERO=SDF('T',CRITU,DFVAP,DELTA);
```

```
RETURN(POWERO);FINISH;

MINNVEC=J(1,G,3.1);
CONM=(MINNVEC||{. .})//J(1,G+2,.)//(CVEC||{-1}||COST);
OPTN={1 0};

CALL NLPNRA(RC,XRT,"PT",MINNVEC,OPTN,CONM);
A=T(-1:2);MAT=J(4##G,G,0);
DO I=1 TO G;Z=1;M=J(4,G,1);M[,I]=A;DO J=1 TO
G;Z=Z@M[,J];END;MAT[,I]=Z;END;
MINVEC=FLOOR(XRT);
PVEC=J(NROW(MAT),1,0);NMAT=J(NROW(MAT),G,0);
DO I=1 TO NROW(MAT);
NVEC=MINVEC+MAT[I,];NMAT[I,]=NVEC;
NT=SUM(NVEC);
VARPSI=(LVEC##2)*(VARVEC/NVEC)`;
DELTA=PSID/SQRT(VARPSI);
KV=(LVEC##2)#VARVEC/NVEC;V1=SUM(KV)##2;
V2=SUM((KV##2)/(NVEC-1));DFVAP=V1/V2;
CRITT=TINV(1-ALPHA/2,DFVAP);
POWERT=CDF('T',-CRITT,DFVAP,DELTA)+SDF('T',CRITT,DFVAP,DELTA);
PVEC[I,1]=POWERT;
END;
TVEC=NMAT*CVEC`;
LOC=LOC(TVEC<=COST);
N2MAT=NMAT[LOC,];P2VEC=PVEC[LOC,];T2VEC=TVEC[LOC,];
MAXI=P2VEC[<:>,1];MAXN=N2MAT[MAXI,];
TCMAX=T2VEC[MAXI,];POMAX=P2VEC[MAXI,];
OPTIMAL_N=MAXN;TOTAL_N=SUM(MAXN);MAX_POWER=POMAX;
FIXED_COST=TCMAX;
PRINT 'TWO-SIDED TEST' MAX_POWER[FORMAT=8.4]
FIXED_COST[FORMAT=10.2] OPTIMAL_N TOTAL_N;

CALL NLPNRA(RC,XRO,"PO",MINNVEC,OPTN,CONM);
A=T(-1:2);MAT=J(4##G,G,0);
DO I=1 TO G;Z=1;M=J(4,G,1);M[,I]=A;DO J=1 TO
G;Z=Z@M[,J];END;MAT[,I]=Z;END;
MINVEC=FLOOR(XRO);
PVEC=J(NROW(MAT),1,0);NMAT=J(NROW(MAT),G,0);
```

```
DO I=1 TO NROW(MAT);
NVEC=MINVEC+MAT[I,];NMAT[I,]=NVEC;
NT=SUM(NVEC);
VARPSI=(LVEC##2)*(VARVEC/NVEC)`;
DELTA=PSID/SQRT(VARPSI);
KV=(LVEC##2)#VARVEC/NVEC;V1=SUM(KV)##2;
V2=SUM((KV##2)/(NVEC-1));DFVAP=V1/V2;
CRITL=TINV(ALPHA,DFVAP);CRITU=TINV(1-ALPHA,DFVAP);
IF DELTA<0 THEN POWERO=CDF('T',CRITL,DFVAP,DELTA);
ELSE POWERO=SDF('T',CRITU,DFVAP,DELTA);
PVEC[I,1]=POWERO;
END;
TVEC=NMAT*CVEC`;
LOC=LOC(TVEC<=COST);
N2MAT=NMAT[LOC,];P2VEC=PVEC[LOC,];T2VEC=TVEC[LOC,];
MAXI=P2VEC[<:>,1];MAXN=N2MAT[MAXI,];
TCMAX=T2VEC[MAXI,];POMAX=P2VEC[MAXI,];
OPTIMAL_N=MAXN;TOTAL_N=SUM(MAXN);MAX_POWER=POMAX;
FIXED_COST=TCMAX;
PRINT 'ONE-SIDED TEST' MAX_POWER[FORMAT=8.4]
FIXED_COST[FORMAT=10.2] OPTIMAL_N TOTAL_N;
QUIT;
```
